# Correlation between retinal sensitivity assessed by microperimetry and structural abnormalities on optical coherence tomography after successful epiretinal membrane surgery

**DOI:** 10.1186/s40942-024-00542-8

**Published:** 2024-02-29

**Authors:** Aline Mota Freitas Matos, Raphael Lucas Sampaio Defina, Luciana Virgínia Ferreira Costa-Cunha, Leandro Cabral Zacharias, Rony Carlos Preti, Mário Luiz Ribeiro Monteiro, Leonardo Provetti Cunha

**Affiliations:** 1 Division of Ophthalmology, Federal University of Juiz de Fora Medical School, Avenida Barão do Rio Branco, 4051. Bom Pastor, Juiz de Fora, Minas Gerais 36021-630 Brazil; 2 Juiz de Fora Eye Hospital, Juiz de Fora, Minas Gerais Brazil; 3 Division of Ophthalmology and the Laboratory of Investigation in Ophthalmology (LIM 33), University of São Paulo Medical School, São Paulo, Brazil

**Keywords:** Disorganization of the retinal inner layers/DRIL, Dissociated optic nerve fiber layer/DONFL, Epiretinal membrane, Macula, Microcysts, Microperimeter/microperimetry, Optical coherence tomography, Outer retinal changes, Pars-plana posterior vitrectomy, Retina

## Abstract

**Background:**

To verify the correlation between retinal sensitivity (RS) assessed by the microperimetry (MP) and optical coherence tomography (OCT) parameters measured in eyes submitted to pars-plana vitrectomy (PPV) for idiopathic epiretinal membrane (ERM) treatment.

**Methods:**

43 patients underwent PPV. Best-corrected visual acuity (BCVA) and OCT imaging were acquired preoperatively and 6 months after surgery. The RS values were recorded 6 months after the surgery. Total macular thickness (TMT) measurements and OCT-evaluated structural findings were also analyzed. The MP examination tested 44 points, with direct topographic correspondence with the OCT-ETDRS map. Correlations between BCVA, RS, and OCT parameters were assessed.

**Results:**

TMT measurements in patients were significantly thicker preoperatively and reduced after surgery. All patients demonstrated BCVA improvements after surgery. The RS parameters after surgery were significantly lower in patients. For OCT structural analyses, patients with lower RS at the fovea correlated with the preexisting disorganization of retinal inner layers (DRIL). In addition, lower RS values were associated with DRIL, outer retinal changes (ORC), and intraretinal microcysts after surgery.

**Conclusions:**

The RS values after surgery were significantly lower when compared to controls. The DRIL presence before and after surgery, and microcysts and ORC after surgery were related to worse visual outcomes.

## Background

Macular epiretinal membrane (ERM) is characterized by the growth of fibrocellular tissue on the retina’s surface, where tangential tractional forces are generated, leading to macular constriction and thickening [1]. Pars-plana vitrectomy (PPV) and ERM peeling are currently the standard of care for visual improvement [2, 3]. However, persistent visual complaints, such as visual blurring, scotomas, and metamorphopsia, are common despite successful anatomical surgery, possibly related to persistent retinal structural changes [4].

Optical coherence tomography (OCT) is the main diagnostic tool for assessing ERM structural changes in pre-and postoperative periods [5]. OCT is used to estimate disease severity, chances for visual recovery and to assess the retina status after surgery. As demonstrated in previous studies, the total macular thickness measurements correlate with the disease’s severity and the magnitude of the visual loss after the surgery [6, 7]. In addition to OCT thickness analysis, previous studies have investigated other biomarkers that could impact visual recovery in ERM cases, such as disorganization of the retinal inner layers (DRIL), intraretinal microcysts, outer retinal changes, or dissociated optic nerve fiber layer (DONFL) [7–10].

The correlation between OCT structural changes and visual function is widely used to understand the mechanisms related to visual loss in patients with ERM and to estimate the likelihood of postoperative visual recovery [11]. This correlation is mainly done by assessing the best corrected visual acuity (BCVA). However, many other psychophysical tests, such as contrast sensitivity, color vision, and standard automated perimetry (SAP), can bring additional insights for evaluating visual function in ERM patients [12].

In this scenario, the microperimetry test may be an alternative method for evaluating macular diseases [13, 14]. The MP-3 microperimeter (Nidek Technologies, Padua, Italy) is a new modality that promotes objective and quantitative retinal sensitivity (RS) measurements with promising applications for macular diseases. [11, 13, 15]. The MP evaluates the macular sensitivity combined with a fundus image, allowing a more direct correlation between RS and the tested area. The assessment of RS in the macular area and its correlation with OCT structural changes can bring new insights into understanding the visual recovery after ERM surgery [11, 15–17].

The purpose of this study was to verify the correlation between the RS assessed by the MP and the qualitative and quantitative parameters measured by the swept-source (SS) OCT in eyes submitted to PPV for the idiopathic ERM treatment.

## Methods

### Study design

An observational, prospective study included patients undergoing PPV to remove ERM with internal limiting membrane (ILM) peeling. Patients with visual loss and metamorphopsia were included. To avoid the influence of media opacity on the RS responses, we selected only pseudophakic patients. We include patients with ages ranging from 50 to 85 years; refractive errors between 5 sphere and three cylindric diopters; preoperative BCVA between 20/25 and 20/200; IOP ≤ 21 mmHg. We selected age-matched healthy controls to compare the macular thickness parameters before and after surgery and microperimetry RS results 6 months after surgery.

The following exclusion criteria were per or postoperative complications; previous history of rhegmatogenous retinal detachment, trabeculectomy, and complicated cataract surgery; previous intravitreal injections; phakic eyes; corneal opacity; glaucoma or other optic neuropathies, diabetic retinopathy, vascular occlusions; axial diameter greater than 25 mm; systemic diseases, except for well-controlled systemic arterial hypertension.

All patients underwent a complete ophthalmological examination before and at months 1, 3, and 6 after surgery. The complete eye exam was performed. The BCVA measurements were assessed using a Snellen chart and converted to a logarithm of the minimum angle of resolution units (log MAR) for statistical analyses. BCVA tests and OCT data were collected before and after surgery, and the MP exam was performed 6 months after surgery.

In all patients, a 25-gauge PPV was performed with a 7500 cpm vitrectomy probe (Constellation Vision System, Alcon). The ERM and ILM were simultaneously stained by Membrane Blue Dual (D.O.R.C., Netherlands). The ERM was grasped and peeled with Eckardt End gripping forceps followed by the ILM peeling. All patients were operated on by the same surgeon (L.P.C) at Juiz de Fora Eye Hospital.

### Optical coherence tomography

Patients underwent OCT examination before and at 1, 3, and 6 months postoperatively. SS-OCT high-resolution B-scan sectional and volumetric images covering up to 7 × 7 mm of the macular area with a scan density of 512 × 256 were acquired.

All images were reviewed for artifacts generated during acquisition or segmentation errors. If these had occurred, the images were discharged, and a new acquisition was performed.

**Fig. 1:**
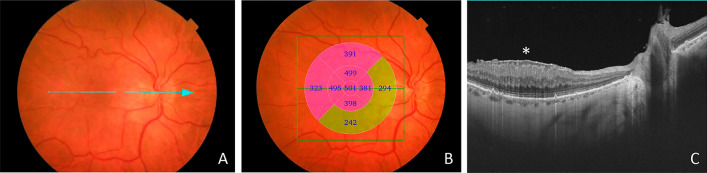
Representative images of a patient with an idiopathic epiretinal membrane before the surgery. **A**: Fundus image showing the ERM in the macular area. The blue arrow represents the OCT-scanned area through the center of the macula. **B**: Fundus image of the same patient with the OCT total macular thickness measurements according to the division into nine sectors of the ETDRS map. **C**: The cross-sectional OCT image showing the ERM (asterisk) with the thickening of the macula

The total macular thickness (TMT) measurements analysis was performed according to the division into nine sectors of the ETDRS-map (Fig. 1).

**Fig. 2:**
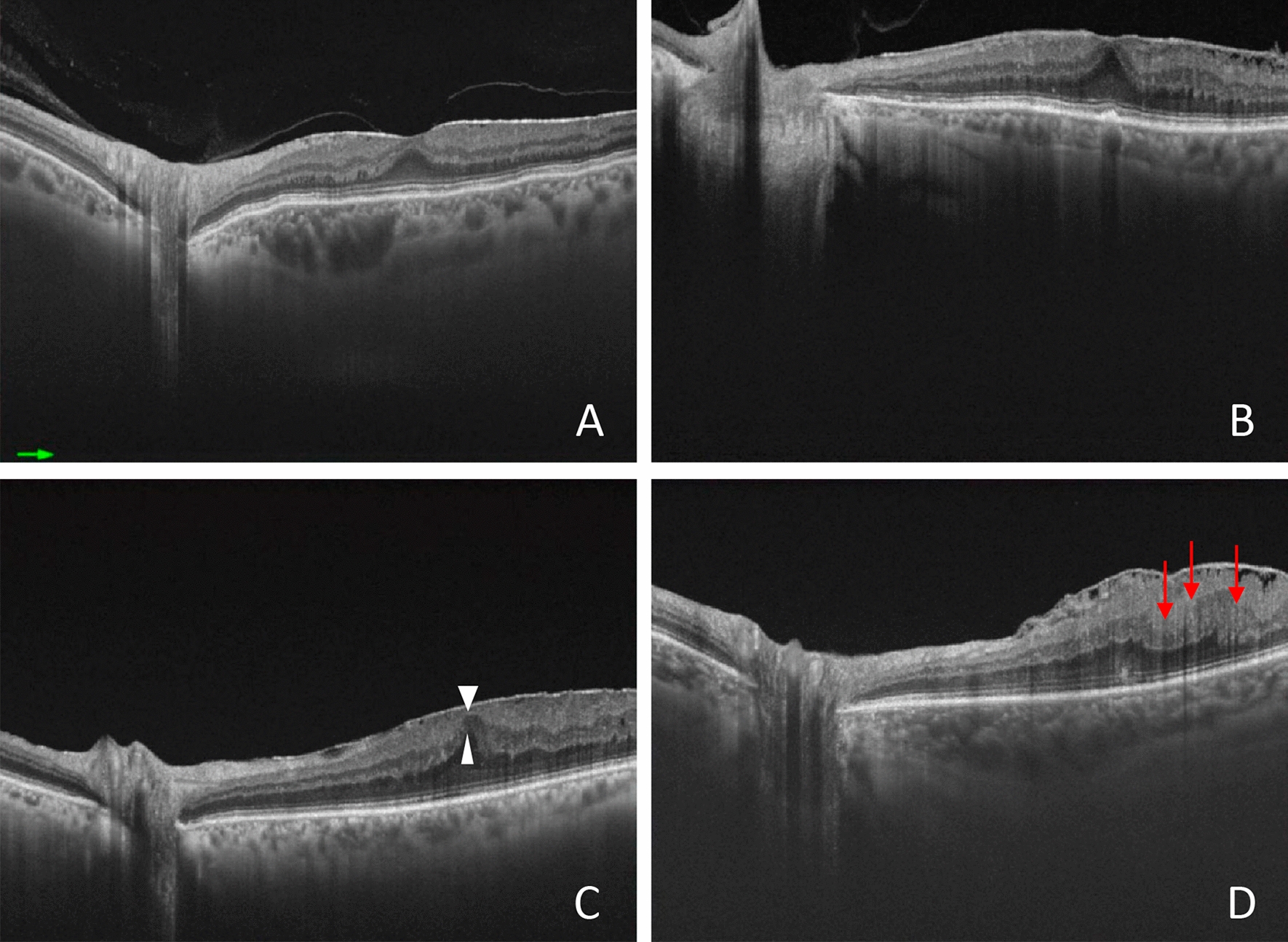
Representative swept-source optical coherence tomography images taken according to the stage system. **A**: Stage 1—negligible morphological or anatomical disruption, retinal layers, and foveal pit are identified. **B**: Stage 2—characteristic stretching of the outer nuclear layer, absence of foveal depression, retinal layers are identified. **C**: Stage 3—continuous ectopic inner foveal layers (white triangles) crossing the central foveal area, absence of foveal depression, retinal layers are identified. **D**: Stage 4—significant retinal thickening, remarkable anatomical disruption of the macula, continuous ectopic inner foveal layers crossing the entire foveal area, retinal layers are significantly distorted (DRIL, red arrows), and the foveal pit is absent

The ERM OCT findings were graded in 1–4 stages [18], depending on the absence of foveal depression, presence of ectopic inner foveal layers (EIFL) and DRIL (Fig. 2). The EIFL was defined as the presence of a continuous hypo or hyper-reflective band extending from the inner nuclear layer and inner plexiform layer over the fovea [18]. According to this classification, in stage 1, the foveal depression is present, and the retinal layers are well-defined. In stage 2, the foveal depression is absent, but the retinal layers are well-defined. In stage 3, the foveal pit is absent and there is additionally EIFL presence, but all retinal layers are clearly identified. In stage 4, the foveal pit is absent, and EIFL and DRIL were presented (Fig. 2). Stage 1 ERM patients were not included in the study.

**Fig. 3:**
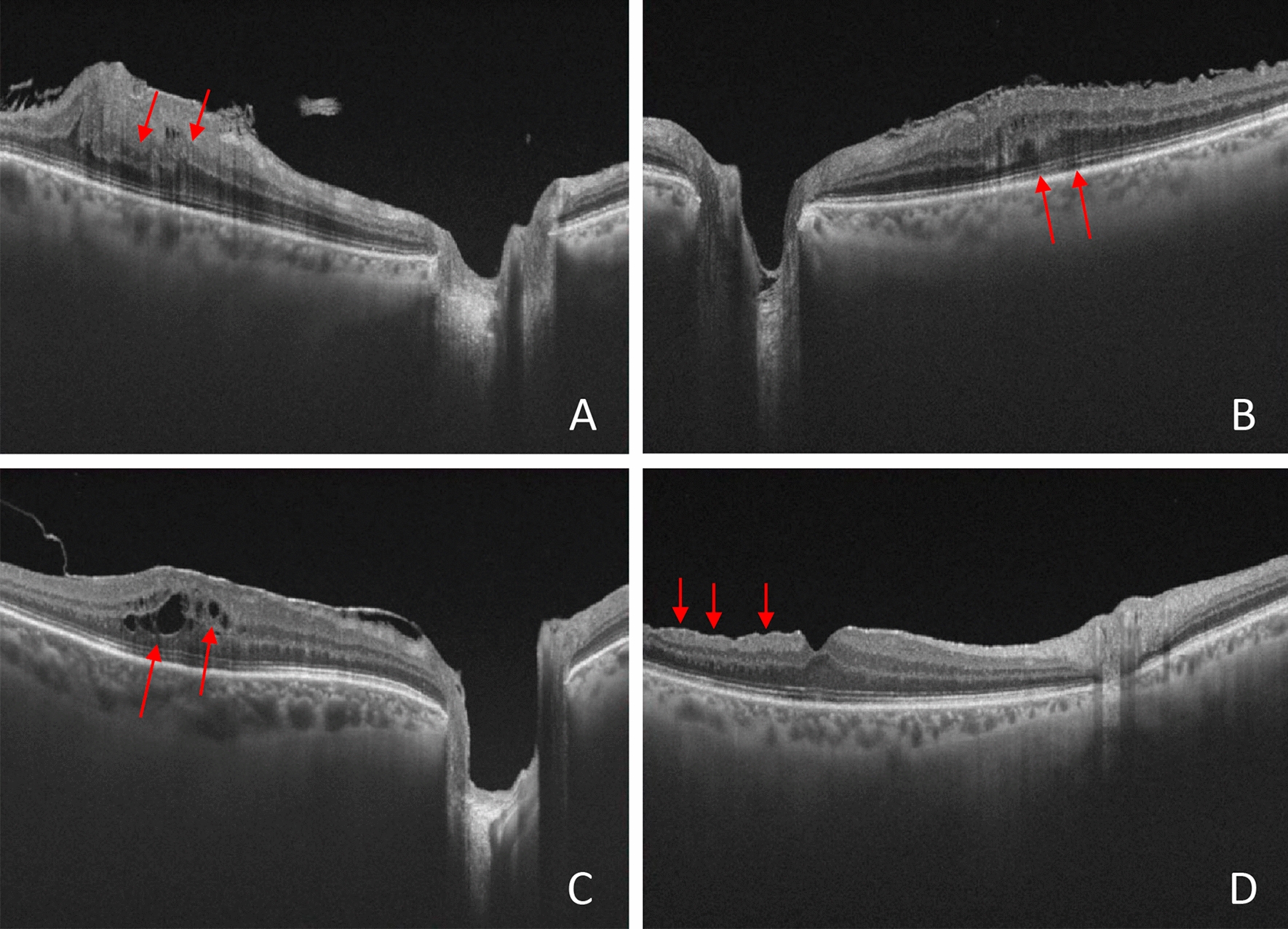
Representative swept-source optical coherence tomography images taken in patients with epiretinal membrane before the surgery (**A**–**C**) and before the surgery (**D**). A: Note the presence of the disorganization of retinal inner layers (DRIL, red arrows). DRIL was defined as the horizontal extent in microns for which the boundaries between the ganglion cell, inner plexiform, and outer nuclear plexiform layers cannot be identified on OCT images. **B**: Representative image of outer retinal changes (red arrows), showing the discontinuities and interruptions of the ellipsoid zone (EZ) and external limiting membrane (ELM) at OCT B-scans images. **C**: Representative image of intraretinal microcystic spaces, which was defined as the presence of dark, hyporreflective cystic spaces located within inner nuclear, Henle’s fiber, or outer plexiform layers (red arrows). **D**: Dissociated optic nerve fiber layer (DONFL, red arrows) stands for striated pattern caused by small dimples at the surface of the inner retina where the internal limiting membrane (ILM) had been removed, visualized at OCT scan images

SS-OCT scans were performed 6 months after surgery, and TMT was recorded. The two high-resolution B-scans passing through the center of the fovea in the vertical and horizontal directions were analyzed for the presence of the following structural changes (Fig. 3): 1. DRIL; 2. irregularities and interruptions of the external limiting membrane (ELM) and ellipsoid zone (EZ), labeled as outer retinal changes(ORC); 3. presence of intraretinal microcysts; 4. presence of dissociated optic nerve fiber layer (DONFL) (Fig. 3). DRIL was defined as the disorganization of retinal inner layers and is the horizontal extent in microns for which the boundaries between the ganglion cell, inner plexiform, and outer nuclear plexiform layers cannot be identified on OCT images [19]. Intraretinal microcystic spaces are defined as dark, hyporreflective cystic spaces within inner nuclear, Henle’s fiber, or outer plexiform layers [20]. ORC were defined as discontinuities and interruptions of the EZ and ELM at OCT images [4]. DONFL stands for striated pattern caused by small dimples at the surface of the inner retina where the ILM had been removed [8].

Two independent examiners graded and evaluated OCT-structural changes. The degree of agreement between them was assessed using Cohen’s Kappa test for agreement analysis. When there was disagreement between the examiners, a third party was consulted.

### Microperimetry test

**Fig. 4:**
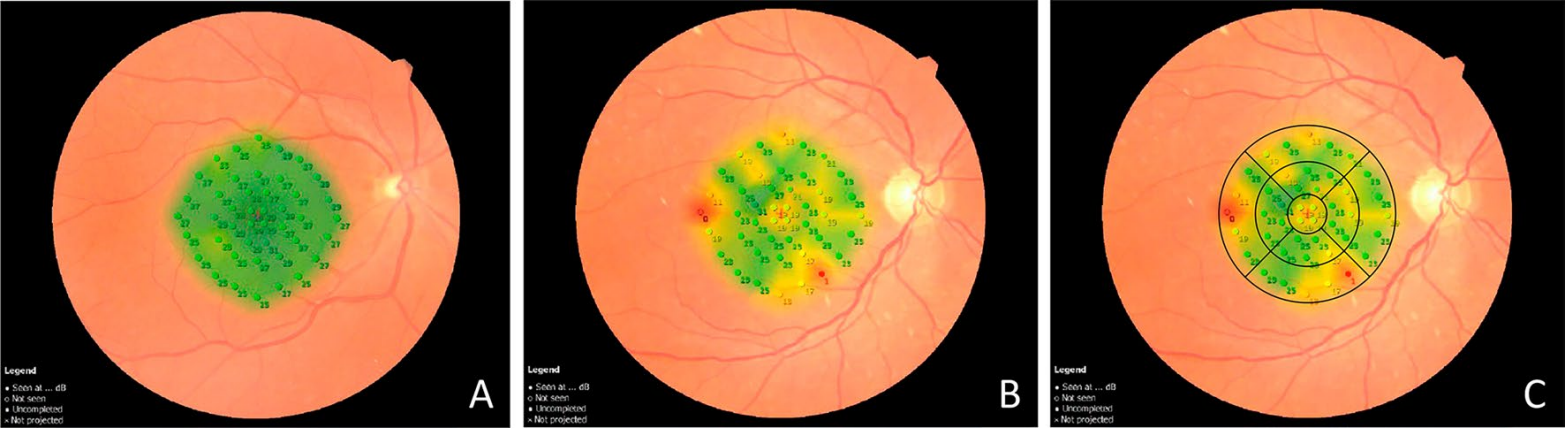
Representative images of microperimetry (MP) test. The exam combines a fundus camera image and microperimetry grid overlaid, with 44 tested points covering 20 central degrees (10 degrees from the center of the fovea in each direction), covering a 6 mm diameter at the macular area. **A**: The representation of MP in the normal control eye. The green color represents retinal sensitivity (RS) response within normal limits. **B**: Example of MP test in a patient after ERM surgery. Note the presence of areas of RS responses within normal limits (green), borderline (yellow), and outside normal limits (red). **C**: The same patient is represented in B, with the OCT ETDRS map with a direct topographic projection over the MP-tested area. Each inner and outer ETDRS map sector's thickness measurements covered by the OCT contain five RS-tested points, while in the central circle (1 mm), there are four RS-tested points

The MP exam was recorded in all patients 6 months after surgery and in control eyes. The MP parameters tested were 44 points covering a total of 20 central degrees, covering a 6 mm in diameter at the macular area with a direct topographic correspondence with the nine sectors of the ETDRS map (Fig. 4). Thus, it was possible to perform a direct topographic correlation between RS MP-tested points and the TMT measurements. Each inner and outer ETDRS map sector contains five RS-tested points, while in the central circle (1 mm), there are four RS-tested points (Fig. 4).

MP stimulus was a Goldmann size III aimed at 200 ms projection time, with a white-back background and a 1.27 CD/m^2^ background luminance, equaling four apostilbs (ASB). The maximum luminance of the MP was 10,000 ASB, and the stimulus attenuation light was programmed between 0 dB, which represents the maximum luminance of the stimulus, and 34 dB, which represents minimal stimulus luminance. If the stimulated area could not notice the maximum visual stimulus threshold, this area was defined as an absolute scotoma (0 dB). A 4–2 threshold strategy (Full-Threshold Staircase) was used. The MP test includes an eye-tracking system to compensate eye movements and monitor the fixation. All exams were performed after mydriasis. First, a pretest with two consecutive MP tests was performed to improve test reliability. The exam was conducted after a 15 min rest period. The device software automatically calculated the mean average of all 44 total threshold point measurements (in dB) for each patient, corresponding to the mean RS. The mean RS responses were also calculated in each of the nine sectors of the ETDRS map.

### Statistical analysis

The Mcnemar test was used to compare a proportion of category variables before and after surgery. The Chi-Square test was used to compare proportions between patient and control groups. Prospects of normality and equality of variations were evaluated by the Komolgorov-Smirnov test and the Levene test, respectively. Pearson’s correlation coefficient was calculated to assess continuous variables. Cohen Kappa coefficient of agreement determined inter-observer agreement for qualitative variables and ERM classification. The t-test for an independent sample was used to compare MP and OCT parameters between patients and controls. The paired t-test was used to compare the differences between parameters before and after surgery. An analysis of variance (ANOVA) was used to test differences in BCVA according to ERM presence with the Bonferroni posthoc test. Receiver operating characteristic (ROC) curves were used to evaluate the ability of OCT parameters to discriminate patients from controls. Finally, multiple linear regression was used to determine predictors of visual acuity after surgery. All analyzes were made in IBM SPSS Statistics (version 22.0; IBM Corporation).

## Results

A total of 43 patients aged between 52 and 84 years (mean 69.4 ± 4.4 years) met the inclusion criteria and were followed for a mean period of 9.6 ± 6.6 months after the surgery. In addition, 43 age- and sex-matched healthy individuals were selected as control group. Table 1 presents the clinical characteristics of participants of the study. There were no statistically significant differences between groups regarding gender, age, and intraocular pressure. There was a statistically significant difference in the BCVA of patients and controls in the pre and postoperative period (p < 0.001 and 0.03, respectively). Almost half of the patients were stage 3. (Table 1). The higher prevalence of DRIL before the surgery reduced significantly after surgery (p = 0.007). The DONFL was only observed after surgery.

**Table 1:** Clinical characteristics of patients and controls

Variables	Category/	Patients	Controls	p-value
	Measure	(n = 43)	(n = 43)	
Age (years)	–	69.4 ± 4.4	68.3 ± 7.9	0.45^a^
Gender	Women	24 (55.8%)	24 (55.8%)	1.00^b^
	Men	19 (44.2%)	19 (44.2%)	
Visual acuity (LogMAR)	Pre-op	0.37 ± 0.19	0.01 ± 0.04	** < 0.001**^a^
	Post-op	0.04 ± 0.09	0.01 ± 0.04	**0.03**^a^
	** < 0.001**^d^			
IOP		14.6 ± 3.5	13.6 ± 2.4	0.12^a^
ERM classification	1	0 (0.0%)		
	2	12 (27.9%)		
	3	21 (48.8%)		
	4	10 (23.3%)		
Time after surgery(months)		9.6 ± 6.6		
OCT findings				
DRIL		18 (41.9%)		
*Preoperative*				
*Postoperative*		7 (16.3%)		
Outer retinal changes				**0.007**^c^
*Preoperative*		11 (25.6%)		
*Postoperative*		10 (23.3%)		
Intraretinal microysts				1.00^c^
*Preoperative*		12 (27.9%)		
*Postoperative*		15 (34.9%)		
DONFL *Postoperative*		38 (88.4%)		0.58^c^

Bold values denote statistical significance at the p < 0.05

*IOP* intraocular pressure, *ERM* epiretinal Membrane, *DRIL* disorganization of the retinal inner layers, *OCT* Optical coherence tomography, *DONFL* dissociated optic nerve fiber layer

^a^Independent t-test

^b^Chi-square test

^c^McNemar test

^d^Paired t-test (BCVA pre versus postoperative)

Inter-observer agreement was high for qualitative OCT variables, including ERM classification and pre (i.e., DRIL presence) and postoperative findings (i.e., DRIL, ORC, microcysts, and DONFL presence). Absolute agreement (%) and Cohen's Kappa coefficient (r) for each of the variables were, respectively: ERM classification (93.3%; r = 0.88); preoperative DRIL (90.0%; r = 0.80); postoperative DRIL (96.6%; r = 0.89); ORC (93.3%; r = 0.79); microcysts (93.3%; r = 0.85); and DONFL (86.6%; r = 0.43). 

Table 2 shows the TMT measurements. In all patients, TMT measurements were significantly higher before the surgery. After surgery, there was a significant reduction of TMT in all sectors (p < 0.001). Postoperative TMT measurements remained higher than controls in the four inner sectors and the fovea.

**Table 2:** Mean values of pre- and postoperative total macular thickness measurements (in µm) obtained by OCT in patients and controls, divided into nine sectors plus average thickness and macular volume, with the respective values of the areas under the ROC curve

OCT total macular thickness (µm)	Patients (n = 43)	Controls (n = 43)	p-value	AUC
Fovea				
Pre-op	455.4 ± 67.2		< 0.001^a^	0.99 (0.98–1.00)
Post-op	373.5 ± 58.7	241.1 ± 34.3	< 0.001^a^	0.96 (0.91–1.00)
p-value	< 0.001^a^			
Temporal inner				
Pre-op	419.9 ± 58.1		< 0.001^a^	0.99 (0.97–1.00)
Post-op	328.9 ± 56.8	298.1 ± 16.1	0.001^a^	0.73 (0.62–0.84)
p-value	< 0.001^a^			
Superior inner				
Pre-op	427.9 ± 54.1		< 0.001^a^	0.99 (0.98–1.00)
Post-op	344.5 ± 35.1	309.8 ± 17.1	< 0.001^a^	0.83 (0.75–0.92)
p-value	< 0.001^a^			
Nasal inner				
Pre-op	410.7 ± 52.8		< 0.001^a^	0.94 (0.89–1.00)
Post-op	358.5 ± 36.2	310.6 ± 17.9	< 0.001^a^	0.89 (0.81–0.96)
p-value	< 0.001^a^			
Inferior inner				
Pre-op	397.1 ± 55.3		< 0.001^a^	0.93 (0.88–0.99)
Post-op	334.7 ± 31.5	308.2 ± 20.5	< 0.001^a^	0.76 (0.66–0.86)
p-value	< 0.001^a^			
Temporal outer				
Pre-op	312.5 ± 51.8		< 0.001^a^	0.90 (0.83–0.98)
Post-op	259.2 ± 27.1	254.4 ± 11.5	0.30	0.55 (0.43–0.68)
p-value	< 0.001^a^			
Superior outer				
Pre-op	327.0 ± 42.7		< 0.001^a^	0.91 (0.84–0.98)
Post-op	279.0 ± 29.4	269.4 ± 14.6	0.06	0.64 (0.52–0.76)
p-value	< 0.001^a^			
Nasal outer				
Pre-op	328.4 ± 39.6		< 0.001^a^	0.88 (0.80–0.95)
Post-op	286.9 ± 26.9	284.5 ± 15.2	0.61	0.53 (0.41–0.66)
p-value	< 0.001^a^			
Inferior outer				
Pre-op	297.9 ± 45.5		< 0.001^a^	0.76 (0.65–0.88)
Post-op	258.7 ± 24.7	259.8 ± 13.5	0.80	0.50 (0.38–0.63)
p-value	< 0.001^a^			
Average thickness				
Pre-op	342.8 ± 33.6		< 0.001^a^	0.97 (0.92–1.00)
Post-op	289.1 ± 23.1	275.1 ± 13.3	0.001^a^	0.71 (0.60–0.82)
p-value	< 0.001^a^			
Macular volume				
Pre-op	9.7 ± 0.9		< 0.001^a^	0.97 (0.92–1.00)
Post-op	8.2 ± 0.6	7.8 ± 0.4	0.001^a^	0.71 (0.60–0.82)
p-value	< 0.001^a^			

*OCT* Optical coherence tomography, *AUC* area under the ROC (receiver operating characteristic) curve

^a^Represents p < 0.05, by paired Student’s t-test (pre- versus post-surgery) and for independent samples (patients versus controls), pre-op: preoperative, post-op: postoperative

Regarding MP findings, after the surgery, the RS was significantly lower in patients for all sectors and for the mean retinal sensitivity. The best parameter performance for the MP was the RS at the fovea (AUC = 0.89) (Table 3).

**Table 3:** Mean values of retinal sensitivity in decibel (dB) measurements obtained by postoperative microperimetry (MP) divided into 9 sectors plus the mean sensitivity, with the respective values of the areas under the ROC curve

Retinal sensitivity (dB)	Patients (n = 43)	Controls (n = 43)	p-value	AUC
Fovea	21.6 ± 4.3	27.1 ± 2.8	< 0.001*	0.89 (0.81–0.96)
Temporal inner	24.0 ± 3.2	27.6 ± 1.5	< 0.001*	0.85 (0.76–0.94)
Superior inner	23.1 ± 3.5	26.8 ± 1.8	< 0.001*	0.84 (0.74–0.93)
Nasal inner	22.9 ± 4.3	26.9 ± 1.9	< 0.001*	0.80 (0.69–0.91)
Inferior inner	23.5 ± 2.7	26.5 ± 1,9	< 0.001*	0.82(0.72–0.92)
Temporal outer	23.6 ± 3.4	26.7 ± 1.8	< 0.001*	0.81 (0.71–0.91)
Superior outer	21.9 ± 3.5	25.9 ± 2.2	< 0.001*	0.85 (0.75–0.94)
Nasal outer	21.9 ± 4.7	21.9 ± 4.7	< 0.001*	0.81(0.71– 0.91)
Inferior outer	21.7 ± 3.6	25.4 ± 2.3	< 0.001*	0.82 (0.72–0.92)
Mean sensitivity	22.7 ± 3.3	26.5 ± 1.8	< 0.001*	0.85 (0.76–0.94)

*dB* decibel

^*^Represents p < 0.05, by umpaired Student's t-test for independent samples (patients versus controls)

Significant differences in the BCVA were observed before surgery, correlated to the ERM stage, and for the presence of ORC (p = 0.02) and intraretinal microcystic (p = 0.01) before the surgery (Table 4). The preoperative BCVA was worse in patients with ERM stage 4 and those with preoperative intraretinal microcysts (p = 0.02) and pre and postoperative ORC (p = 0.03 and 0.04, respectively). A significant correlation was found between the ERM stage (2, 3, and 4) and BCVA before the surgery (r = 0.29, 0.36 and 0.51, respectively p = 0.02) (Table 4).

**Table 4:** Mean ± standard deviation of postoperative retinal sensitivity assessed by microperimetry and its correlation with epiretinal membrane (ERM) classification and pre and postoperative optical coherence tomography findings

	Postoperative microperimetry			
	(em dB)			
	Fovea	p-value	Mean sensitivity	p-value
ERM classification				
2	23.0 ± 3.4		23.4. ± 2.8	
3	22.7 ± 5.4	0.33^a^	23.4 ± 4.4	0.56^a^
4	20.4 ± 4.9		21.6 ± 5.8	
Preoperative DRIL				
Yes	19.8 ± 4.5	**0.006**^**b**^	21.2 ± 5.4	**0.046**^**b**^
No	23.8 ± 4.4		23.5 ± 3.2	
Preoperative outer retinal changes				
Yes	20.1 ± 5.6	0.11	21.3 ± 6.5	0.30
No	22.9 ± 4.4		23.5 ± 3.2	
Preoperative intraretinal microcysts				
Yes	20.7 ± 4.1	0.23	22.9 ± 3.0	0.78
No	22.9 ± 4.1		23,4 ± 3.7	
Postoperative DRIL				
Yes	18.5 ± 3.9	**0.03**^**b**^	21.6 ± 3.2	0.27
No	22.6 ± 3.2		23.3 ± 3.0	
Postoperative outer retinal changes				
Yes	18.7 ± 4.8	**0.007**^**b**^	19.1 ± 6.3	**0.001**^**b**^
No	23.2 ± 4.4		23.4 ± 3.3	
Postoperative intraretinal microcysts				
Yes	19.3 ± 5.0	**0.003**^**b**^	20.3.7 ± 5.7	**0.02**^**b**^
No	23.6 ± 2.9		23.9 ± 3.3	
DONFL				
Yes	21.9 ± 5.0	0.26	22.8 ± 4.6	0.49
No	24.5 ± 2.4		24.3 ± 1.1	

Bold values denote statistical significance at the p < 0.05

*ERM* epiretinal membrane, *DRIL* disorganization of the retinal inner layers, *OCT* optical coherence tomography, *DONFL* dissociated optic nerve fiber layer

^a^ANOVA test

^b^p values < 0.05 obtained by Student’s t test for independent samples

There was a positive correlation between preoperative BCVA and the postoperative foveal thickness (r = 0.42, p = 0.005). In addition, a negative correlation was observed between preoperative BCVA with mean foveal sensitivity after surgery (r = − 0.38, p = 0.01). The worse preoperative BCVA was related to the lower RS values at the fovea after surgery. Our results demonstrated a significant correlation between RS values and TMT parameters postoperatively in the temporal and superior outer sectors (p = 0.03 and 0.04, respectively).

The preoperative DRIL correlates with the mean sensitivity and the RS at the fovea (p = 0.006 and 0.046, respectively). Similarly, postoperative DRIL correlates with the RS at the fovea (p = 0.03). The ORC and intraretinal microcysts after surgery correlate with the RS at the fovea (p = 0.007 and 0.003, respectively) and the mean sensitivity (p = 0.001 and 0.002, respectively) (Table 5).

**Table 5:** Mean ± standard deviation of postoperative retinal sensitivity assessed by microperimetry and its correlation with epiretinal membrane (ERM) classification and pre and postoperative optical coherence tomography findings

	Postoperative microperimetry			
	(em dB)			
	Fovea	p-value	Mean sensitivity	p-value
ERM classification				
2	23.0 ± 3.4		23.4. ± 2.8	
3	22.7 ± 5.4	0.33^a^	23.4 ± 4.4	0.56^a^
4	20.4 ± 4.9		21.6 ± 5.8	
Preoperative DRIL				
Yes	19.8 ± 4.5	**0.006**^b^	21.2 ± 5.4	**0.046**^b^
No	23.8 ± 4.4		23.5 ± 3.2	
Preoperative outer retinal changes				
Yes	20.1 ± 5.6	0.11	21.3 ± 6.5	0.30
No	22.9 ± 4.4		23.5 ± 3.2	
Preoperative intraretinal microcysts				
Yes	20.7 ± 4.1	0.23	22.9 ± 3.0	0.78
No	22.9 ± 4.1		23,4 ± 3.7	
Postoperative DRIL				
Yes	18.5 ± 3.9	**0.03**^b^	21.6 ± 3.2	0.27
No	22.6 ± 3.2		23.3 ± 3.0	
Postoperative outer retinal changes				
Yes	18.7 ± 4.8	**0.007**^b^	19.1 ± 6.3	**0.001**^**b**^
No	23.2 ± 4.4		23.4 ± 3.3	
Postoperative intraretinal microcysts				
Yes	19.3 ± 5.0	**0.003**^b^	20.3.7 ± 5.7	**0.02**^**b**^
No	23.6 ± 2.9		23.9 ± 3.3	
DONFL				
Yes	21.9 ± 5.0	0.26	22.8 ± 4.6	0.49
No	24.5 ± 2.4		24.3 ± 1.1	

Bold values denote statistical significance at the p < 0.05

*ERM* epiretinal membrane, *DRIL* disorganization of the retinal inner layers, *OCT* optical coherence tomography, *DONFL* dissociated optic nerve fiber layer

^a^ANOVA test

^b^p values < 0.05 obtained by Student's t test for independent samples

In the multivariate analysis, the mean RS at the fovea was considered the dependent variable. The presence of DRIL before the surgery was the main related variable. On average, the mean RS at the fovea was 4.6 dB lower after surgery in patients with preoperative DRIL.

## Discussion

Our results demonstrated that all patients showed significant visual acuity (VA) improvement 6 months after the surgery. Previous studies demonstrated that preoperative BCVA was an important prognostic factor for final BCVA [21, 22]. Patients with the best BCVA before surgery had better postoperative results. Regarding ERM classification, the higher the stage, the worse BCVA was before surgery [18]. The correlation between membrane stage and preoperative BCVA can be explained by the greater macular distortion. Similarly, Goveto et al. [18] demonstrated that more advanced ERM stages were associated with lower VA. Another interesting finding was that BCVA before surgery correlates with foveal thickness after surgery. On the other hand, the BCVA after surgery did not correlate with any macular thickness parameters. Following our results, Lee et al. [23] demonstrated that preoperative foveal thickness correlates with visual improvement after ERM surgery.

We found reduced RS parameters in all patients. Similarly, despite the great visual improvement, the BCVA remains worse after surgery when compared to the control eyes. However, the significance level of these two parameters, RS and BCVA after surgery (p < 0.001 and p = 0.03, respectively), was greater for the RS, with the AROC curve ≥ 0.80 in all sectors. These findings suggests that RS assessments can be a more sensitive indicator of macular disfunction after ERM surgery [15, 17, 24]. Accordingly, no correlation was found between postoperative BCVA and any OCT thickness measurements. Conversely, the ORC before surgery were the only OCT parameter correlating with BCVA postoperatively.

Our results demonstrated that many OCT parameters correlate with MP values. The presence of DRIL (before and after surgery), ORC, and intraretinal microcysts after surgery correlated RS values. Zur et al. [9] were the first to explore the predictive value of DRIL in ERM patients. Eyes with the presence of preoperative DRIL experienced the worst visual outcomes [9]. The DRIL-related worst prognosis is secondary to continuous mechanical traction, deformation of the inner retinal layers, distortion, and disruption of synapses between photoreceptors and ganglion cells. Karasavvidou et al. [6] confirmed these findings, demonstrating that TMT and severe DRIL were related to worse BCVA. However, in our study, pre or postoperative DRIL did not correlate with the final BCVA. DRIL was associated with the mean RS at the fovea before and after surgery. This finding suggests that DRIL may be a predictive biomarker of worse visual function recovery. Furthermore, preoperative DRIL was the main variable responsible for predicting the RS values at the fovea in the logistic regression analysis.

Another structural finding in OCT that correlates with the RS at the foveal area was the presence of intraretinal microcysts after surgery. Lee et al. [10] also demonstrated that microcystic macular edema in cases of ERM was a significantly poor prognostic factor for visual recovery. The persistent presence of intraretinal cystoid spaces after surgery could be related to chronic structural changes of the macula. Microcystic changes can be frequently observed in other macular diseases, such as age-related macular degeneration, macular hole, or vitreomacular traction, suggesting the blood-retinal barrier breakdown or focal inflammation. Similarly, in optic nerve diseases, such as glaucoma, optic neuritis, and chiasmal compression, the microcysts in the inner nuclear layer can also be observed [20, 25, 26]. In these cases, retrograde transsynaptic degeneration is probably the causative factor, and cystic spaces are commonly located at the inner nuclear layer [10]. Some authors believe that microcysts are related to Müller cell dysfunction [20]. So, it is possible that in ERM patients, both inflammatory and degenerative mechanisms are present. In more chronic cases, the microcysts’ presence may result from a degenerative process, while in early postoperative periods, the inflammatory cause may be more likely. Previous studies failed to demonstrate that cyst treatment results in visual improvement [10]. Therefore, eyes without intraretinal cystoid spaces seem to be associated with better visual recovery [27].

The ORC in the postoperative period were another biomarker related to worse RS responses. Previous studies have demonstrated that ORC are a common finding in more advanced cases of ERM. EZ and ELM disruption often reflect irreversible damage to photoreceptors and are associated with poorer visual outcomes [28].

The presence of DONFL was the most frequent ultrastructural OCT change after surgery, present in 38 of 43 cases. This finding is associated with the ILM peeling [8, 29]. In our study, the presence of DONFL did not correlate with either visual acuity or postoperative RS values. Blautain et al. [29] and Arias et al. [30] showed that ILM peeling after ERM surgery was unrelated to worse visual outcomes. Likewise, in the present study, there was no correlation between DONFL and MP parameters.

Our study has limitations, such as a relatively small sample size and not having a more prolonged follow-up. Since all patients were submitted to ERM surgery concomitant to ILM peeling, we could not compare the outcomes in patients where the ILM was not peeled. Our study’s strength was correlating MP parameters with OCT macular thickness measurements in a more direct topographical analysis. To our knowledge, this is the first study to perform this specific form of analysis.

## Conclusion

In summary, all patients submitted to ERM surgery demonstrated visual acuity improvement 6 months after surgery. However, the RS values assessed by MP were significantly lower when compared to control eyes. We showed that some biomarkers could be related to worse visual outcomes after ERM surgery, including worse preoperative BCVA, ERM severity, and the presence of some OCT structural changes such as DRIL before and after surgery, as well for the presence of intraretinal microcysts and ORC after surgery. We believe that RS parameters assessed by MP can bring additional information and may help to understand better the correlations between structural and functional findings after ERM surgery.

## Data Availability

The datasets generated during and/or analyzed during the current study are available from the corresponding author on reasonable request.

## Abbreviations

RS: Retinal sensitivity
OCT: Optical coherence tomography
PPV: Pars-plana vitrectomy
BCVA: Best-corrected visual acuity
TMT: Total macular thickness
ETDRS: Early treatment diabetic retinopathy study
ERM: Macular epiretinal membrane
DRIL: Disorganization of the retinal inner layers
ORC: Outer retinal changes
DONFL: Dissociated optic nerve fiber layer
SAP: Standard automated perimetry
MP: Microperimetry
SS: Swept-source
ILM: Internal limiting membrane
IOP: Intraocular pressure
mmHg: Millimetre of mercury
mm: Milimetre
logMAR: Logarithm of the minimum angle of resolution
cpm: Cuts per minute
EIFL: Ectopic inner foveal layers
EZ: Ellipsoid zone
ELM: External limiting membrane
ASB: Apostils
dB: Decibel
ANOVA: Analysis of variance
AROC: Area under receiver operating characteristic curve
VA: Visual acuity
